# Outcomes following planned two-stage exchange arthroplasty for periprosthetic joint infections in the United States: a systematic review of the literature

**DOI:** 10.1007/s00402-025-05955-0

**Published:** 2025-06-23

**Authors:** Nicolas Piuzzi, Larry Yost, William Putnam, Bryan Springer, Brian de Beaubien, Kenneth Urish, Javad Parvizi

**Affiliations:** 1 Department of Orthopaedic Surgery, Cleveland Clinic, Cleveland, USA; 2 The Atticus Group, Portsmouth, USA; 3 Osteal Therapeutics, Dallas, USA; 4 Department of Orthopedic Surgery, Mayo Clinic, Rochester, USA; 5 Department of Orthopedics, Covenant Medical Center, Lubbock, USA; 6 Department of Orthopaedic Surgery, University of Pittsburgh, Pittsburgh, USA; 7 Arthritis and Arthroplasty Design Group, The Bone and Joint Center, UPMC Magee-Womens Hospital, Pittsburgh, USA; 8 International Joint Center, Acıbadem University, Istanbul, Turkey

**Keywords:** Periprosthetic joint infection, Two-stage, Total knee arthroplasty, Total hip arthroplasty

## Abstract

**Introduction:**

While two-stage exchange arthroplasty is the gold standard for treating periprosthetic joint infections (PJIs), it results in a prolonged treatment period with the potential for complications and non-planned additional procedures, limited joint function during the interstage period, and emotional stress for patients. The primary objective of this systematic literature review was to evaluate outcomes associated with two-stage exchange arthroplasty for treating total hip arthroplasty (THA) and total knee arthroplasty (TKA) PJIs. This literature review analyzed U.S. data on the timing and health consequences associated with the interstage period and outcomes following reimplantation in patients undergoing two-stage exchange arthroplasty.

**Materials and Methods:**

A search of U.S. studies published between January 2014 and January 2024 was conducted using PubMed and Embase databases.

**Results:**

Sixty-five studies reporting data on 26,354 patients undergoing two-stage exchange arthroplasty were included. There were 29.0% and 68.6% of patients who underwent THA and TKA respectively, with 2.4% patients not having the affecting joint identified. The mean interstage period was 141.4 ± 74.2 days with 16.9 ± 12.2% patients not re-implanted. The mean infection eradication was 74.2 ± 10.5% and the average reinfection rate was 15.7 ± 7.1%. Complications and additional procedures were common during the interstage and post-implant periods. Only four studies utilized Musculoskeletal Infection Society (MSIS) Outcomes Reporting Tool. Patients undergoing treatment for TKA PJIs had less successful MSIS outcomes compared to those being treated for THA PJIs. This includes a lower rate of infection control (46.0% vs. 65.5%), a higher rate of reoperation, revision, and/or spacer retention (40.6% vs. 25.2%) and a higher death rate (13.4% vs. 9.4%), respectively.

**Conclusion:**

Two-stage exchange arthroplasty treatment of PJIs is associated with major morbidity and often requires additional surgical procedures to address complications. The prolonged duration of the interstage period contributes to morbidity and negatively impacts patients’ quality of life and increases the risk of mortality.

## Introduction

Periprosthetic joint infections (PJIs) are a serious complication that can occur after total knee arthroplasty (TKA) and total hip arthroplasty (THA) leading to noteworthy morbidity, mortality, prolonged treatment, and often require revision surgery [1]. PJIs occur in approximately 1–2% of primary TKA and THA procedures with an even higher risk following revision surgeries [2–4]. Patient-related risk factors associated with the development of PJIs include diabetes mellitus, obesity, rheumatoid arthritis, immunosuppression, malnutrition, and prior joint infections [5–9]. Perioperative risk factors associated with PJIs include prolonged operative time, intraoperative contamination, poor surgical technique, hematomas, wound complications and lack of appropriate antibiotic prophylaxis [10]. 

Treatment strategies for PJIs include (1) debridement, antibiotics, and implant retention (DAIR) for early acute infections with stable implants, (2) one-stage exchange arthroplasty which involves the removal of the infected prosthesis, debridement, and implantation of a new prosthesis in the same surgery followed by a period of antibiotic therapy, (3) two-stage exchange arthroplasty with the removal of the infected prosthesis, placement of an antibiotic spacer and a period of antibiotic therapy followed by implantation of a new prosthesis at a second surgery, (4) removal of the prosthesis without replacement and a period of antibiotic therapy for non-ambulatory patients or those with significant comorbidities or (5) amputation and a period of antibiotic therapy in patients with a severe, life-threatening infection where other treatments have failed. The gold-standard for PJI treatment is currently two-stage exchange arthroplasty [11]. 

PJIs impose a substantial economic burden due to extended hospitalizations, multiple surgeries, and prolonged antibiotic therapy. A recent analysis reported the median total direct costs at 2 years for patients undergoing two-stage exchange arthroplasty for PJIs was $38,865 for reimplantation alone and $79,223 for those undergoing reimplantation with revision [12]. By 2023, annual hospital costs for hip and knee PJI in the U.S. are projected to be an estimated $1.85 billion [13]. Additionally, the prolonged interstage period associated with two-stage exchange arthroplasty also has a significant negative impact on both the physical and mental aspects of a patient’s patient quality of life (QoL) [14, 15]. During the interstage period, patients have limited mobility, increased dependency on others and worsened mental health, and potential complications such as spacer-related issues, wound healing problems, and adverse reactions to antibiotics can further impact QoL.

The primary objective of this systematic review was to evaluate clinical outcomes associated with two-stage exchange arthroplasty for the treatment of PJIs in the United States. Specifically, we analyzed data on interstage timing, reimplantation rates, infection eradication, reinfection rates, complications, and additional procedures, with a comparison of outcomes for THA versus total knee arthroplasty TKA PJIs.

## Methods

### Literature search and inclusion criteria

A systematic literature review was conducted to identify relevant publications related to the treatment of total hip arthroplasty (THA) and total knee arthroplasty (TKA) related periprosthetic joint infections (PJIs) in the United States. The literature review was performed in line with PRISMA (Preferred Reporting Items for Systematic Reviews and Meta-Analyses) guidelines [16]. The literature search was conducted on January 5, 2024, using PubMed and EMBASE databases using the search terms listed in Table 1. The search was limited to publications in the English language between January 1, 2014, and January 5, 2024. Original studies reporting clinical outcomes of patients who underwent two-stage exchange arthroplasties of the hip or knee with at least fifty patients were considered eligible for this analysis. Case reports, technical notes, abstracts, editorial commentaries, ex-vivo, and pre-clinical studies (on animal or cadavers) were excluded.

**Table 1:** Systematic search terms

	Search Terms	Search Results	
		PubMed	Embase
1	(((((prosthetic joint infection[Title/Abstract]) OR (periprosthetic joint infection[Title/Abstract])) OR (PJI[Title/Abstract])) OR (prosthesis-related infections[Title/Abstract])) OR (prosthesis infection[Title/Abstract]))	6,552	7,252
2	#1 AND (total knee arthroplasty OR TKA OR total knee OR total hip arthroplasty OR THA OR revision knee OR revision hip)	3,771	2,889
3	#2 AND (2 stage OR 2-stage OR two-stage OR two-stage)	1,081	654
4	#3 AND ENGLISH[Language]	1,052	646
5	#4 AND (“2014“[Date - Entry]: “2024“[Date - Entry])	988	599
6	#5 AND “Clinical Trial” OR “Randomized Controlled Trial” OR “Clinical Study”	150	493
7	#6 AND ([conference abstract]/lim OR [conference paper]/lim OR [conference review]/lim OR [editorial]/lim OR [letter]/lim OR [review]/lim)	0	53
8	#6 NOT #7	150	440
9	#8 - Total combined with duplicates removed	525	

**Fig. 1:**
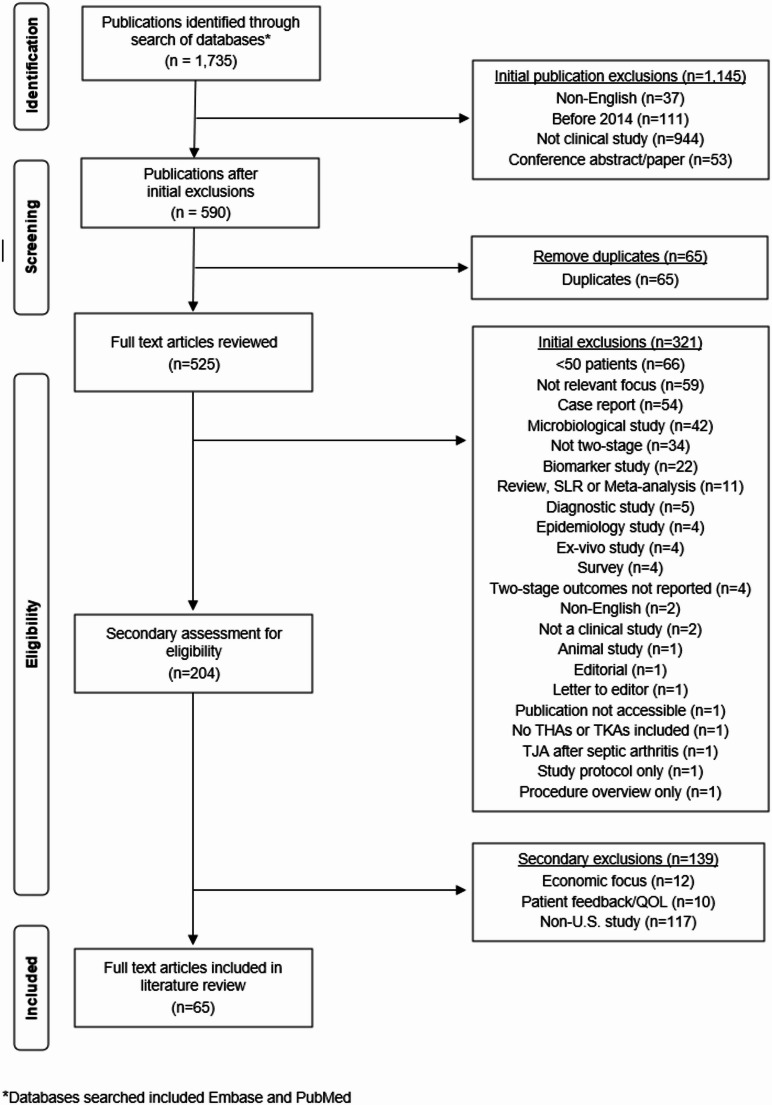
PRISMA Flowchart (Preferred Reporting Items for Systematic Reviews and Meta-Analyses) guidelines demonstrating the publication selection process for the systematic literature review of the treatment of total hip and total knee arthroplasty periprosthetic joint infections in the United States

The study selection process is shown in Fig. 1. A total of 525 publications were available for full-text review after the initial search, removal of duplicates, and assessment by an independent reviewer. Following full-text review, non-relevant publications and publications which reported on case series of fewer than 50 patients were removed. Non-U.S. studies reporting clinical outcomes following two-stage treatment of THA and TKA PJIs were also removed due to the potential for variations in clinical practices outside of the United States. The final literature review following full text review is based on the remaining 65 publications.

### Endpoints and statistical analysis

The primary endpoints for this analysis included (1) the duration of interstage period defined as the mean number of days between the initial stage of the two-stage procedure and reimplantation; (2) reimplantation success defined as the percentage of patients who successfully underwent reimplantation following the first stage of the two-stage procedure; (3) infection eradication assessed as the rate of infection eradication achieved following reimplantation; (4) reinfection rate defined as the percentage of patients who experience reinfections following initial treatment; (5) complications during the interstage period; and (6) the frequency and types of additional procedures performed during the interstage period and after reimplantation. Study methodology, patient age and gender, and affected joint were also extracted and tabulated. Continuous variables were reported as non-weighted means and standard deviations. Categorical variables were reported as number of patients and percentages. Statistical analysis was conducted using Microsoft Excel for Microsoft 365 MSO (Version 2409 Build 1 6.0.18025.20030) 64-bit (Microsoft Corporation, Redmond, WA, USA).

## Results

### Study characteristics

Table 2 reports on the characteristics of the 65 studies included in the primary analysis [17–81]. This included 17 (26.2%) studies that reported on PJIs following THA, 26 (40.0%) studies reporting on PJIs following TKA, and 22 (33.8%) studies reporting outcomes following both THA and TKA. Several of the studies reporting on both THAs and TKAs did not differentiate outcomes by the affected joint. There were only 5 (7.7%) studies that were prospective, with each of these having a randomized, controlled study design. The remaining 60 (92.3%) studies were retrospective, with 34 of the 60 (56.7%) having a comparative arm and the remaining 26 (43.3%) being single-arm studies.

**Table 2:** Patient and study characteristics

Author	Year	Joint	Study Type	Study Design	Total Patients	Age (years)	Males/ Females	2-Stage Hips	2-Stage Knees
Houdek MT, et al. [17]	2015	Hip	Retrospective	Single arm	99	63 ± 10	32/57	99	0
Cancienne JM, et al. [18]	2017	Hip	Retrospective	Single arm	7,146	All patients > 65 years of age	NR	4,301	0
Chalmers BP, et al. [19]	2018	Hip	Retrospective	Single arm	131	65 (35–91)	76/55	135	0
Goel R, et al. [20]	2018	Hip	Retrospective	Single arm	297	62.9 (24.3–88.0)	135/162	222	0
Jones CW, et al. [21]	2019	Hip	Retrospective	Comparative	185	64 ± 13.6	107/78	170	0
Petis SM, et al. [22]	2019	Hip	Retrospective	Single arm	162	68.3 ± 11 (34–90)	106/56	164	0
Tirumala V, et al. [23]	2021	Hip	Retrospective	Comparative	138	1 stage: 68.88 ± 9.47 2 stage: 68.17 ± 8.28	45/47	92	0
Dagneaux L, et al. [24]	2021	Hip	Retrospective	Single arm	227	65 (21–89)	85/142	256	0
Kerbel YE, et al. [25]	2021	Hip	Retrospective	Comparative	50	60.2 *±* 10.3	27/23	50	0
Li K, et al. [26]	2021	Hip	Retrospective	Single arm	205	65	97/106	189	0
Nahhas CR, et al. [27]	2021	Hip	Prospective	Randomized	52	58.2 ± 10.4	41/11	40	0
Lancaster AJ, et al. [28]	2021	Hip	Retrospective	Comparative	58	59.8 ± 10.7	23/35	49	0
Debbi EM, et al. [29]	2022	Hip	Retrospective	Comparative	All patients: 99 Ceramic-on-polyethylene: 32 Cement on bone: 67	All patients: 68.4 Ceramic-on-polyethylene: 67.0 ± 11.1 Cement on bone: 69.1 ± 12.5	54/45	89	0
Kugelman D, et al. [30]	2022	Hip	Retrospective	Comparative	All patients: 104 Cement spacer: 75 Real component spacer: 29	All patients: 65.4 Cement spacer: 63.4 ± 11.7 Real component spacer: 67.0 ± 10.2	103/61	89	0
Nace J, et al. [31]	2023	Hip	Retrospective	Comparative	123	1.5 stage: 61 (33–88) Two-stage: 59 (31–80)	35/34	69	0
Whittaker MJ, et al. [32]	2023	Hip	Retrospective	Comparative	All patients:76 PJI: 49 Aseptic revision: 27	PJI: 62 (32–86) Aseptic revision: 68 (35–88)	30/19	49	0
Sharqzad AS, et al. [33]	2019	Hip	Retrospective	Comparative	274	63.6 + 12.4	45/45	90	0
Watts CD, et al. [34]	2014	Knee	Retrospective	Comparative cohort	All patients: 111 Morbidly obese: 37 Non-obese: 74	morbidly obese: 60 ± 9 years non-obese: 62 ± 8 years	33/78	0	111
Sabry FY, et al. [35]	2014	Knee	Retrospective	Comparative cohort	314	65 years (36–88)	165/149	0	291
Brimmo O, et al. [36]	2016	Knee	Retrospective	Comparative	All patients: 750 Prior I&D: 57 No prior I&D: 693	over 65: 311 (41%) under 65: 439 (59%)	358/392	0	All patients: 750 Prior I&D: 57 No prior I&D: 693
Lichstein P, et al. [37]	2016	Knee	Retrospective	Single arm	107	67 (42–89)	53/54	0	107
Nodzo SR, et al. [38]	2017	Knee	Retrospective	Comparative	All patients: 140	All patients: 66.2	90/50	0	140
Cancienne JM, et al. [39]	2018	Knee	Retrospective	Single arm	18,533	0	NR	0	11,420
Petis SM, et al. [40]	2019	Knee	Retrospective	Single arm	245	0	123/122	0	245
Siddiqi A, et al. [41]	2019	Knee	Retrospective	Comparative	193	Single-stage < 60 y 14 (24.6%) 60–74 y 28 (49.1%) >75 y 15 (26.3%) Two-stage < 60 y 56 (40.9%) 60–74 y 59 (43.1%) > 75 y 22 (16.1%)	79/115	0	136
Zielinski MR, et al. [42]	2019	Knee	Retrospective	Comparative	Total: 85 Articulating spacers without IM dowels 49 (57.7%) Articulating spacers with IM dowels 14 (16.5%) Static spacers with IM dowels 22 (25.9%)	66 ± 9.0	49/36	0	85
Nahhas CR, et al. [43]	2020	Knee	Prospective	Randomized, Controlled	49	65.3	26/23	0	49
Dagneaux L, et al. [44]	2021	Knee	Retrospective	Single arm	424	67 years (31–92)	211/213	0	455
Woon CYL, et al. [45]	2021	Knee	Retrospective	Comparative	140	Not reported	80/60	0	140
Roof MA, et al. [46]	2021	Knee	Retrospective	Comparative	Total: 164 All cement 72 (43.9%) Real component 92 (56.1%)	All cement: 63.35 ± 11.73 Real component: 66.98 ± 10.17	84/80	0	126
Barry JJ, et al. [47]	2021	Knee	Retrospective	Comparative	87	DAIR: 68.9 ± 13.0 Two-stage: 66.3 ± 8.7	43/44	0	31
Klemt C, et al. [48]	2021	Knee	Retrospective	Comparative cohort	132	One stage: 65.1 ± 9.4 Two-stage: 65.9 ± 8.8	48/40	0	88
Huffaker SJ, et al. [49]	2022	Knee	Retrospective	Comparative cohort	1410	DAIR: 68.7 *±* 9.8 Two-stage: 68.5 *±* 8.9	198/212	0	410
Buller LT, et al. [50]	2022	Knee	Retrospective	Comparative	103	67.2 *±* 8.8	66/37	0	103
Charalambous LT, et al. [51]	2022	Knee	Retrospective	Single arm	55	64.5 *±* 11.5	28/27	0	30
Nabet A, et al. [52]	2022	Knee	Retrospective	Comparative	162	1.5 stage: 64 ± 10.9 Two-stage: 62 ± 11	20/28	0	48
Christiner T, et al. [53]	2022	Knee	Retrospective	Single arm	146	68.5 ± 9.3	74/70	0	146
Kildow BJ, et al. [54]	2022	Knee	Retrospective	Multi-center, Single arm	178	66.5	87/91	0	165
Shichman I, et al. [55]	2023	Knee	Retrospective	Multi-center, Single arm	90	65 (23–92)	53/37	0	72
Belay ES, et al. [56]	2023	Knee	Retrospective	Comparative	116	1.5 stage:66 (31–85) Two-stage: 65 (45–81)	33/25	0	58
Oladipo VA, et al. [57]	2023	Knee	Retrospective	Comparative	391 Functional spacer (*n* = 336) Non-functional spacer (*n* = 55)	67.1 (34.7–93.4)	197/194	0	391
Klemt C, et al. [58]	2023	Knee	Retrospective	Comparative	All patients (*n* = 198) Normalized ESR and CRP (*n* = 96) Elevated ESR and CRP (*n* = 21) Elevated ESR and normalized CRP (*n* = 47) Normalized ESR and elevated CRP (*n* = 34)	Normalized ESR and CRP: 65.6 *±* 8.4 Elevated ESR and CRP: 65.2 *±* 8.7 Elevated ESR and normalized CRP: 64.6 *±* 8.5 Normalized ESR and elevated CRP: 65.3 *±* 8.3	102/96	0	198
Hartzler MA, et al. [59]	2020	Knee	Retrospective	Single arm	134	66.9 *±* 8.7	69/65	0	123
Dietz MJ, et al. [60]	2014	Knee & Hip	Retrospective	Comparative	All patients (*n* = 54) Transfer (*n* = 18) No transfer (*n* = 36)	Transfer: 65.3 (43–88) No transfer: 64.9 (39–83)	27/27	Total patients (*n* = 18) Transfer (*n* = 6) No transfer (*n* = 12)	All patients (*n* = 36) Transfer (*n* = 12) No transfer (*n* = 24)
Gomez MM, et al. [61]	2015	Knee & Hip	Retrospective	Comparative	504	0	NR	137	280
Siqueira MB, et al. [62]	2015	Knee & Hip	Retrospective	Comparative	All patients (*n* = 368) Chronic antibiotic suppression (*n* = 92) No antibiotic suppression (*n* = 276)	Chronic antibiotic suppression: 63.7 ± 11.7 No antibiotic suppression: 64.2 ± 11.5	49/88	162* Chronic antibiotic suppression (*n* = 38) No antibiotic suppression (*n* = 124)	162* Chronic antibiotic suppression (*n* = 38) No antibiotic suppression (*n* = 124)
Kheir MM, et al. [63]	2017	Knee & Hip	Retrospective	Single arm	60	Not reported	NR	40*	40*
Kheir MM, et al. [64]	2017	Knee & Hip	Retrospective	Single arm	87	Not reported	NR	43*	43*
Frank JM, et al. [65]	2017	Knee & Hip	Prospective	Multicenter, randomized	107	64 *±* 10	66/41	50	57
Geller JA, et al. [66]	2017	Knee & Hip	Retrospective	Single arm	247	64 (24–93)	119/128	156	91
Tan TL, et al. [67]	2018	Knee & Hip	Retrospective	Single arm	409	65	211/198	127	282
Aali Rezaie A, et al. [68]	2018	Knee & Hip	Retrospective	Single arm	282	65.6 ± 10.8 years	142/140	95	187
Wouthuyzen-Bakker M, et al. [69]	2019	Knee & Hip	Retrospective	Comparative	344	Not reported	210/134	138	206
Barton CB, et al. [70]	2020	Knee & Hip	Retrospective	Single arm	89	64 years (43–84)	50/37	47	14
Borsinger TM, et al. [71]	2021	Knee & Hip	Retrospective	Comparative cohort	121	66 *±* 10	57/64	39	63
Klemt C, et al. [72]	2021	Knee & Hip	Retrospective	Comparative cohort	All patients (*n* = 245) Spacer exchange (*n* = 49) No spacer exchange (*n* = 196)	Total patients: 66.5 Spacer exchange: 65.8 ± 8.7 No spacer exchange: 66.7 ± 9.8	163/82	Total patients (*n* = 108) Spacer exchange (*n* = 20) No spacer exchange (*n* = 88)	Total patients (*n* = 137) Spacer exchange (*n* = 29) No spacer exchange (*n* = 108)
van den Kieboom J, et al. [73]	2022	Knee & Hip	Retrospective	Comparative cohort	All patients (*n* = 120) Infected internal fixation (*n* = 40) PJI (*n* = 80)	Total patients: 64.1 ± 13.3 Infected internal fixation: 63.2 ± 14.4 PJI: 64.1 ± 13.3	65/55	Total patients (*n* = 82) Infected internal fixation (*n* = 27) PJI (*n* = 55)	Total patients (*n* = 38) Infected internal fixation (*n* = 13) PJI (*n* = 25)
Gabrielli AS, et al. [74]	2022	Knee & Hip	Retrospective	Comparative	All patients (*n* = 240) No sinus tract (*n* = 188) Sinus tract (*n* = 52)	No sinus tract: 62.0 ± 11.1 Sinus tract: 65.0 + 11.3	123/117	Total patients (*n* = 70) No sinus tract (*n* = 53) Sinus tract (*n* = 17)	Total patients (*n* = 170) Sinus tract (*n* = 135) No sinus tract (*n* = 35)
Valenzuela MM, et al. [75]	2022	Knee & Hip	Prospective	Randomized	All patients (*n* = 127) One-stage (*n* = 66) Two-stage (*n* = 61)	One-stage: 65.8 *±* 9.3 Two-stage: 68.4 ± 8.2	37/27	18	48
Hartman CW, et al. [76]	2022	Knee & Hip	Retrospective	Single arm	158	64.5 + 11.5	80/78	56	102
Metcalf RW, et al. [77]	2023	Knee & Hip	Retrospective	Comparative	All patients (*n* = 470) Males (*n* = 248) Females (*n* = 222)	Total patients: 65.5 *±* 9.9 Males: 65.1 *±* 10.4 Females: 66 *±* 9.3	248/222	Total patients:429* Males: 224* Females: 205*	Total patients:429* Males: 224* Females: 205*
Fehring TK, et al. [78]	2023	Knee & Hip	Retrospective	Single arm	390	Not provided	NR	0	0
Ryan SP, et al. [79]	2023	Knee & Hip	Retrospective	Comparative	All patients (*n* = 444) No Antibiotics (*n* = 102) <2 week antibiotic (*n* = 296) > 2 week antibiotic (*n* = 76)	No Antibiotics: 68 (60–75) <2 week antibiotic: 65 (59–73) > 2 week antibiotic: 66 (61–73)	257/187	Total (*n* = 210) No Antibiotics (*n* = 43) <2 week antibiotic (*n* = 133) > 2 week antibiotic (*n* = 34)	Total (*n* = 234) No Antibiotics (*n* = 59) <2 week antibiotic (*n* = 133) > 2 week antibiotic (*n* = 42)
Van Den Kieboom J, et al. [80]	2021	Knee & Hip	Retrospective	Comparative	105	65.0 *±* 11.0	41/34	27	48
Yang J, et al. [81]	2020	Knee & Hip	Prospective	Multi-center, randomized	All patients (*n* = 142) Culture directed oral antibiotic (*n* = 72) No Antibiotics (*n* = 70)	Culture directed oral antibiotic 62.9 *±* 10.8 No Antibiotics 63.3 *±* 10.5	87/56	63	79

*did not identify whether 2-stage procedures were for THAs vs. TKAs

ETO, extended trochanteric osteotomy; I&D; Incision and drainage; NR, Not reported; PJI, Periprosthetic joint infection; THA, Total hip arthroplasty; TKA, Total knee arthroplasty

### Patient characteristics

A total of 26,354 patients undergoing two-stage exchange arthroplasty were included in the 65 studies (Table 2). The mean age of patients undergoing two-stage procedures, when reported, was similar across most of the studies and 52.0% of patients were male and 48.0% female. There was a total of 7,655 (29.0%) patients with THA PJIs and 18,066 (68.6%) with TKA PJIs. There were 633 (2.4%) patients where the joint was not identified.

### Duration of interstage period

When reported, the mean overall follow-up period varied widely between the studies (*n* = 44) with a non-weighted mean of 43.5 ± 22.4 months (range 4.4 to 101 months) (Table 3). The non-weighted mean number of days between the initial stage (stage 1) of the two-stage procedure and reimplantation (stage 2) for the 31 (47.7%) studies which reported the length of the interstage period was 141.4 ± 74.2 days (range 60.0 to 423 days). For studies that specifically defined the interstage period for hips (*n* = 9) and knees (*n* = 12) alone, the mean interstage periods were 141.4 ± 51.0 days and 127.8 ± 78.3 days, respectively.

**Table 3:** Procedural and outcomes data

Author	Joint	Mean follow-up	Interstage Period	% Failed to Proceed to 2nd Stage	Eradication Two-stage Hip	Reinfection Two-stage Hip	Eradication Two-stage Knee	Reinfection Two-stage Knee
Houdek MT, et al. [17]	Hip	NR	All patients: 29.7 weeks Morbidly Obese: 21 weeks Non-obese: 34 weeks	Not applicable	Not reported	Total: 7 (7.1%) Morbidly Obese: 6 (18.2%) Non-obese: 1 (1.5%)	Not applicable	Not applicable
Cancienne JM, et al. [18]	Hip	NR	124.4 ± 39.3 days	39.8%	Not reported	Not reported	Not applicable	Not applicable
Chalmers BP, et al. [19]	Hip	5 years (2–10)	94 days (40-1104)	Not applicable		8% @ 2 years 12% @ 5 & 10 years	Not applicable	Not applicable
Goel R, et al. [20]	Hip	NR	Not reported	18.7%	Not reported	Not reported	Not applicable	Not applicable
Jones CW, et al. [21]	Hip	78 ± 21 months	14 ± 8.3 weeks	8.1%	113/150 (75.3%)	20 (13%)	Not applicable	Not applicable
Petis SM, et al. [22]	Hip	NR	Not reported	Not applicable	Not reported	9.8% at 1 year, 14.3% at 5 years, and 14.9% at 10 and 15 years	Not applicable	Not applicable
Tirumala V, et al. [23]	Hip	NR	Not reported	Not report	Not reported	14 (15.2%)	Not applicable	Not applicable
Dagneaux L, et al. [24]	Hip	NR	15 weeks (1–87)	Not applicable	Not reported	Not reported	Not applicable	Not applicable
Kerbel YE, et al. [25]	Hip	35.4 months (1–89)	Not reported	Not applicable	Not reported	Total: 7 (14.0%) Morbidly Obese: 6 (33.3%) Obese: 1 (6.3%) Non-obese: 0 (0.0%)	Not applicable	Not applicable
Li K, et al. [26]	Hip	101 months	Not reported	7.8%	140 (69.0%)	23 (12.2%)	Not applicable	Not applicable
Nahhas CR, et al. [27]	Hip	3.2 years	Not reported	7.7%	Not reported	8 (20%)	Not applicable	Not applicable
Lancaster AJ, et al. [28]	Hip	5 years	Not reported	10.9%	36 (73.5%)	Not reported	Not applicable	Not applicable
Debbi EM, et al. [29]	Hip	2.6 years	All patients: 16.37 weeks Ceramic-on-polyethylene: 17.0 ± 13.8 weeks Cement on bone: 16.1 ± 9.8 weeks	10.1%	Not reported	Not reported	Not applicable	Not applicable
Kugelman D, et al. [30]	Hip	All patients: 660.1 days Cement spacer: 902.9 ± 711.7 days Real component spacer: 424.9 ± 402.5 days	All patients: 208.8 days Cement spacer: 188.16 ± 45.50 days Real component spacer: 228.78 *±* 217.21 days	14.4%	Not reported	8 (9.0%)	Not applicable	Not applicable
Nace J, et al. [31]	Hip	1.5 stage: 2.6 years (0.07–6.7) Two-stage: 2.3 years (0.2–8.1)	1.5 stage: 288.35 days Two-stage: 208.05 days	Not applicable	Not reported	12 (17.4%)	Not applicable	Not applicable
Whittaker MJ, et al. [32]	Hip	2.9 years (0.1–12.1)	Not reported	Not applicable	Not reported	10 (20.1%)	Not applicable	Not applicable
Sharqzad AS, et al. [33]	Hip	NR	111.4 ± 83.4 days	Not applicable	Not reported	Not reported	Not applicable	Not applicable
Watts CD, et al. [34]	Knee	morbidly obese: 6.9 years (5.1–10.8) non-obese: 7.9 years (5.0-11.1)	morbidly obese: 73 days (20–355) non-obese: 69 days (18–235)	Not applicable	Not applicable	Not applicable	Not reported	Total: 9.9% Morbidly obese: 21.6% Nonobese 4.1%
Sabry FY, et al. [35]	Knee	NR	103 days (1–2470)	Not applicable	Not applicable	Not applicable	206 (66.6%)	105 (33.4%)
Brimmo O, et al. [36]	Knee	4 years	Not reported		Not applicable	Not applicable	All patients: 624 (83.2%) Prior I&D: 52 (91.3%) No prior I&D: 572 (82.5%)	Not reported
Lichstein P, et al. [37]	Knee	3.7 years (2.0-9.8)	Not reported	Not applicable	Not applicable	Not applicable	109 (93.6%)	Not reported
Nodzo SR, et al. [38]	Knee	NR	All patients:10.7 months PREFAB: 10.7 (7.9–14.5) MOLD: 10.0 (8.0–14.0) AUTOCL: 11.6 (9.4–15.4)	Not applicable	Not applicable	Not applicable	All patients: 90.7% PREFAB: 89.7% MOLD: 95.3% AUTOCL: 87.2%	Not reported
Cancienne JM, et al. [39]	Knee	NR	Not reported	38.4%	Not applicable	Not applicable	Not reported	Not reported
Petis SM, et al. [40]	Knee	NR	Not reported	Not applicable	Not applicable	Not applicable	Not reported	1 year: 4.1% 2 years: 9.5% 5 years: 14.2% 10 years: 16.4% 15 years: 16.9%
Siddiqi A, et al. [41]	Knee	Single-stage: 52.9 ± 4.9 months Two-stage: 54.7 ± 4.1 months	Not reported	Not reported	Not applicable	Not applicable	97 (70.8%)	33 (24.1%)
Zielinski MR, et al. [42]	Knee	NR	2.7 months	Not applicable	Not applicable	Not applicable	71 (83.5%)	Not reported
Nahhas CR, et al. [43]	Knee	3.5 years (2.0-6.4)	Articulating spacer: 76.1 ± 36.9 (60.9–91.3) days Static spacer: 71.5 ± 20.2 (63.0-80.1) days	Not applicable	Not applicable	Not applicable	Not reported	3 (6.1%)
Dagneaux L, et al. [44]	Knee	NR	11 weeks (1–55)	Not applicable	Not applicable	Not applicable	Not reported	Not reported
Woon CYL, et al. [45]	Knee	NR	81.4 days. New spacers (111 days), cement-based spacers (72 days) and autoclaved spacers (84 days).	6.1%	Not applicable	Not applicable	Not reported	Not reported
Roof MA, et al. [46]	Knee	All cement: 902.9 ± 711.7 days Real component: 425.0 *±* 402.5 days	All cement: 188.2 *±* 45.5 days Real component: 228.8 *±* 217.2 days	11.0%	Not applicable	Not applicable	Not reported	14 (11.1%)
Barry JJ, et al. [47]	Knee	DAIR: 3.2 years Two-stage: 3.05 years	6.8 ± 10.3 months (2.5–59.7)	41.9%	Not applicable	Not applicable	67.7%	Not reported
Klemt C, et al. [48]	Knee	NR	74 days (45–123)	Not applicable	Not applicable	Not applicable	Not reported	24 (27.2)%
Huffaker SJ, et al. [49]	Knee	NR	Not reported	Not applicable	Not applicable	Not applicable	Not reported	41 (11.6%)
Buller LT, et al. [50]	Knee	33.5 months	Not reported	Not applicable	Not applicable	Not applicable	Not reported	6 (5.8%)
Charalambous LT, et al. [51]	Knee	29.8 + 16.3 months	Not reported	10.6%	Not applicable	Not applicable	16 (53.3%)	Not reported
Nabet A, et al. [52]	Knee	1 year	0.39 ± 0.35 years	Not applicable	Not applicable	Not applicable	Not reported	12 (25.0%)
Christiner T, et al. [53]	Knee	5.1 ± 0.4 years	17.1 weeks	Not applicable	Not applicable	Not applicable	98 (66.2%)	Not reported
Kildow BJ, et al. [54]	Knee	6.63 years	Not reported	7.3%	Not applicable	Not applicable	152 (85.4%)	1-year: 5.1% 3-year: 11.2% 5-year: 16.9%
Shichman I, et al. [55]	Knee	2.13 years (1.0-10.2)	Not reported	Not applicable	Not applicable	Not applicable	49 (67.8%)	Not reported
Oladipo VA, et al. [57]	Knee	1.5 stage: 32.2 (12.0-82.3) Two-stage: 28.1 months (12.0-100.0)	Not reported	Not applicable	Not applicable	Not applicable	46 (79.3%)	12 (20.7%)
Klemt C, et al. [58]	Knee	All patients: 2.9 years (0.05-13.0) Functional spacer: 3.1 years (0.05-13.0) Non-functional spacer: 1.7 (0.1–6.4)	Not reported	Not applicable	Not applicable	Not applicable	All patients: 85.7% Functional spacer: 85.4% Non-functional spacer: 87.2%	Not reported
Hartzler MA, et al. [59]	Knee	4.4 years (2.8–6.5)	Not reported	Not applicable	Not applicable	Not applicable	Not reported	All patients (24.2%) Normalized ESR and CRP (14.6%) Elevated ESR and CRP (33.3%) Elevated ESR and normalized CRP (27.7%) Normalized ESR and elevated CRP (26.5%)
Dietz MJ, et al. [60]	Knee	NR	Not reported	8.2%	Not applicable	Not applicable	Not reported	30 (24.4%)
Gomez MM, et al. [61]	Knee and Hip	Transfer: 57.5 months (24–120) No transfer: 68.8 months (24–138)	Transfer: 14.1 ± 13.1 months No transfer: 5.2 ± 3.5 months	Not applicable	Total: 36 (66.7%)* Transfer: 8 (44%) No transfer: 28 (78%)	Not reported	Total: 36 (66.7%)* Transfer: 8 (44%) No transfer: 28 (78%)	Not reported
Siqueira MB, et al. [62]	Knee and Hip	All: 56.2 months (1.1-186.9) Knee: 59.7 months (1.9–167.0) Hip: 49.1 months (1.1-186.9)	Not reported	17.3%	89 (81.7%)	Not reported	179 (81.4%)	Not reported
Kheir MM, et al. [63]	Knee and Hip	Chronic immunosuppression: 69.1 ± 38.2 months (2.2-168.3) No suppression: 41.6 ± 40.2 months (1-183)	Chronic immunosuppression: 21.1 ± 9.7 weeks (6.6–46.3) No suppression: 15.0 ± 8.0 weeks (3.1–45.6)	Not applicable	Only reported for patients on chronic antibiotics (*n* = 38)* 25 (65.8%)	Not reported	Only reported for patients on chronic antibiotics (*n* = 38)* 25 (65.8%)	Not reported
Kheir MM, et al [64]	Knee and Hip	NR	Not reported	35% (study population was patients who were reinfected after an initial 2-stage procedure for PJI	16 (62%)*	9 (22.5%)*	16 (62%)*	9 (22.5%)*
Frank JM, et al. [65]	Knee and Hip	NR	Not reported	Not applicable	27 (62.8%)*	Not reported	27 (62.8%)*	Not reported
Geller JA, et al. [66]	Knee and Hip	12 *±* 11 months	Not reported	Not applicable	Not reported	14.0%	Not reported	8.8%
Tan TL, et al. [67]	Knee and Hip	NR	144 days	Not applicable	Not reported	Not reported	Not reported	Not reported
Aali Rezaie A, et al. [68]	Knee and Hip	NR	91.8 days	Not applicable	87.4%	Not reported	83.0%	Not reported
Wouthuyzen-Bakker M, et al. [69]	Knee and Hip	1 year	100.2 days (20–648)	13.6% (includes lost to follow-up)	78 (82.1%)	23 (16.0%)*	141 (75.4%)	23 (16.0%)*
Barton CB, et al. [70]	Knee and Hip	35 months	Not reported	Not applicable	113 (81.9%)	Not reported	167 (81.1%)	Not reported
Borsinger TM, et al. [71]	Knee and Hip	56.3 months	Not reported	31.5% (authors were unable to identify reason for spacer retention in majority of patients due to retrospective nature of the study)	40 (85.1%)	Not reported	12 (85.7%)	Not reported
Klemt C, et al. [72]	Knee and Hip	3.7 *±* 1.7 years	119 days	Not applicable	29 (74.4%)	Not reported	42 (66.7%)	Not reported
van den Kieboom J, et al. [73]	Knee and Hip	2 years	Total patients: 129.2 days Spacer exchange: 191.1 ± 67.2 days No spacer exchange: 113.7 ± 55.4 days	Not applicable	Not reported	Total patients (16.7%)* Spacer exchange (24.5%) No spacer exchange (14.8%)	Not reported	Total patients (16.7%)* Spacer exchange (24.5%) No spacer exchange (14.8%)
Gabrielli AS, et al. [74]	Knee and Hip	4.5 years (1.0-25.8)	Total patients: 105 days Infected internal fixation: 99 days PJI: 108 days	Not applicable	Not reported	Total patients (15.9%) Infected internal fixation (11.1%) PJI (18.2%)	Not reported	Total patients (26.3%) Infected internal fixation (46.2%) PJI (16.0%)
Valenzuela MM, et al. [75]	Knee and Hip	NR	Not reported	16.3%	Not reported	Total patients (18.8%)* No sinus tract (14.4%) Sinus tract (9.6%)	Not reported	Total patients (18.8%)* No sinus tract(14.4%) Sinus tract (9.6%)
Hartman CW, et al. [76]	Knee and Hip	NR	Not reported	Not reported	Not reported	Not reported	Not reported	Not reported
Metcalf RW, et al. [77]	Knee and Hip	3.35 years (2–12)	Successful treatment: 109 days (34–858) Unsuccessful treatment: 141 days (65–363)	Not reported	Not reported	13 (19.6%)*	Not reported	13 (19.6%)*
Fehring TK, et al. [78]	Knee and Hip	1 year	Not reported	Not reported	381 (80%) – reported for all 1 and 2 stage patients together	Not reported	381 (80%) – reported for all 1 and 2 stage patients together	Not reported
Ryan SP, et al. [79]	Knee and Hip	NR	Not reported	1.0%	336 (87.0%)*	50 (13.0%)*	336 (87.0%)*	50 (13.0%)*
Van Den Kieboom J, et al. [80]	Knee and Hip	1 year	Not reported	Not applicable	Not reported	Total 6.7% No Antibiotics 11.6% <2 week antibiotic 6.0% >2 week antibiotic 2.9%	Not reported	Total 8.5% No Antibiotics 13.5% <2 week antibiotic 6.7% >2 week antibiotic 7.1%
Yang J, et al. [81]	Knee and Hip	4.4 years (2.5–22.9)	Not reported	Not applicable	Not reported	3 (11.1%)	Not reported	12 (25.0%)

*did not identify whether 2-stage procedures were for THAs vs. TKAs

NR, not reported

### Failure to undergo reimplantation

Only 21 of 65 (32.3%) studies reported the percentage of patients who had a stage 1 procedure that did not have a stage 2 procedure (Table 4). Reimplantation failure data was not available for most of the studies since they were retrospective and only assessed patients who had completed stage 2 of the two-stage exchange arthroplasty. The non-weighted mean percentage of patients undergoing planned two-stage procedures who were not re-implanted was 16.9 ± 12.2% with 14.7 ± 10.1% of hips and 17.6 ± 14.4% of knees not being reimplanted. Two separate studies by Cancienne et al., which analyzed data from a large claims database, reported that 39.8% and 38.4% of patients with infected THAs and TKAs that had undergone stage 1 of a planned two-stage exchange arthroplasty were not reimplanted within one year of the index procedure [18, 39]. These two studies provide real-world evidence of the true incidence of patients who underwent a stage 1 procedure who, for various reasons, did not go on to reimplantation.

**Table 4:** Post-reimplantation complications and additional procedures

Complication/Procedure	Non-weighted Mean ± SD
Dislocation	6.1 ± 3.3%
Arthrodesis	6.0 ± 4.7%
Amputation	5.2 ± 5.0%
Wound complication	4.4 ± 4.4%
Resection arthroplasty	4.2 ± 4.8%
Aseptic loosening	3.8 ± 2.2%
Instability	3.5 ± 3.9%
Periprosthetic/other fracture	2.9 ± 2.8%
Aseptic revisions/reoperations (reason not defined)	7.0 ± 2.2%
Blood transfusion	36.8 ± 26.7%
Acute kidney injury (AKI)	20.9 ± 1.9%

Cancienne et al. identified a number of risk factors associated with the reduced likelihood of being reimplanted following the stage 1 procedure [18, 39]. Risk factors for not being reimplanted in patients with a PJI following THA were female gender, advanced age, diabetes mellitus, peripheral vascular disease, congestive heart failure, being on hemodialysis, and depression [18]. Risk factors for TKAs were the same plus chronic lung disease [39]. 

### Infection eradication

A total of 27 of 65 (41.5%) studies reported on the percentage of patients for which a two-stage procedure resulted in the successful eradication of PJI as defined by the authors (Table 3). There were 3 studies that only included THAs, 12 studies that only included TKAs, and 12 studies that included both THAs and TKAs. Five of the studies that included both THAs and TKAs did not differentiate outcomes between the two procedures. The non-weighted average eradication rate was 74.2 ± 10.5% across all studies. The average eradication rate across the studies was 73.7 ± 11.0% for THAs and 75.4 ± 10.1% for TKAs.

### Reinfection

A total of 36 of 65 (55.4%) studies reported reinfection rates with either the initial causative organism(s) or new microorganisms following two-stage hip or knee exchange arthroplasty procedures (Table 3). There were 11 studies that included only hips, 14 studies that only included knees, and 14 studies that included both hips and knees. Six of the studies included both but did not differentiate outcomes between the two joints. The non-weighted average reinfection rate was 15.7 ± 7.1% across all studies. The average reinfection rate was 13.3 ± 4.3% for hips and 17.0 ± 9.1% for knees.

### Factors affecting outcomes

Several publications in the present systematic literature review studied individual-specific factors to determine their relationship with outcomes following a two-stage exchange arthroplasty procedure of the hip or knee. Patient-related factors associated with the increased likelihood of unsuccessful eradication of PJI, or an increased risk of reinfection included age, diabetes, chronic kidney disease, immunosuppression, malnutrition and previous joint infections.

Four studies analyzed the relationship between obesity and outcomes, reporting that morbidly obese patients (BMI > 40 kg/m^2^) had a greater rate of reinfections, spacer-related complications and reoperations than non-obese patients (BMI < 30 kg/m^2^)^16,24,33^ and obese patients (BMI > 30 kg/m^2^) had a greater rate of reinfection [20]. 

Aali Rezaie et al. assessed the impact of the time between the stage 1 procedure and stage 2 (reimplantation) on outcomes for patients with chronic PJI of the hip and the knee treated with a two-stage exchange arthroplasty [67]. While time to reimplantation was not significantly associated with failure in both univariate and multivariate models, patients reimplanted at greater than 26 weeks were twice as likely to fail in comparison to those reimplanted at less than 26 weeks (43.8% vs. 21.1%, *p* = 0.057).

Laboratory findings have also been shown to predict treatment success or failure. Klemt et al. reported that elevated serum ESR and/or CRP levels prior to reimplantation in two-stage knee revision surgery for chronic PJI are associated with increased reinfection rates [58]. The presence of specific pathogens was also shown to decrease eradication rates and the likelihood of successful treatment outcomes. These pathogens include Coagulase-negative staphylococci, methicillin-resistant *Staphylococcus aureus* (MRSA), methicillin-resistant *Staphylococcus epidermidis* (MRSE), *Enterococcus* species, vancomycin-resistant enterococci (VRE), and gram-negative bacteria [35, 51, 64, 76]. 

### Antibiotic therapy

There were significant inconsistencies in the reporting of antibiotic regimens across the studies with 20 of the 65 (30.8%) studies not reporting this information (Supplemental Table 1). For the studies which did report on antibiotic regimens used, there were varied approaches to the use of antibiotics during the interstage period with little consistency. Most studies indicated the use of vancomycin with either tobramycin or gentamicin mixed into bone cement and formed into a spacer which is used following implant removal in conjunction with individualized systemic antibiotic therapy based on culture results, most often under the guidance of an infectious disease specialist. Across the studies, there was little consistency in the antibiotic agents used or duration of antibiotic therapy following stage 2 procedures.

Several papers reported on results of studies focusing on the use of differing antibiotic therapy approaches post-reimplantation for patients undergoing two-stage revision procedures for THA or TKA related PJIs. Siqueira et al. reported that chronic oral antibiotics taken for a minimum of six months following reimplantation was associated with increased infection-free prosthetic survival rate for patients that had a *Staphylococcus aureus* infection [62]. In the same study, there was no difference in infection-free rates, however, between the suppression and non-suppression groups following the two-stage revision. Frank et al. reported a reduction in reinfection rates for patients receiving a one-month course of oral antibiotic therapy tailored to the original infecting organisms [64]. Ryan et al. reported patients who had less than 2 weeks of oral antibiotic therapy following reimplantation had fewer reinfections than patients who did not receive oral antibiotic therapy [79]. There was no difference in reinfection rates between individuals receiving less than or more than 2 weeks of antibiotic therapy. Yang et al. conducted a prospective controlled trial where patients were randomized to either receive microorganism-directed oral antibiotics for three months following reimplantation or no antibiotic following hospital discharge [81]. Patients treated with oral antibiotics had significantly fewer PJI treatment failures. Petis et al. reported an increased reinfection rate for patients on antibiotic suppression, noting that suppression was likely used selectively in patients deemed at a high risk for reinfection [22]. They also indicated that 75% of the reinfections were due to a new organism. The above results do not provide conclusive evidence of the benefits of the use of oral antibiotic therapy following two-stage procedures for THA and TKA PJIs.

### Complications and additional procedures

While complications and the need for additional procedures are common during the interstage period and following reimplantation for patients undergoing planned two-stage treatment of infected THAs and TKAs, there were inconsistencies in reporting these for the studies identified for the present literature review. Additionally, none of the studies utilized the Common Terminology Criteria for Adverse Events (CTCAE) to report complications with some publications only reporting selective complications. The limited number of studies which provided detailed information on complications and additional procedures required during the interstage period limits the generalizability of this data.

#### During interstage period

Twenty-five of the 65 (38.5%) studies provided comprehensive or selective information on complications and/or additional procedures performed during the interstage period (Supplemental Table 2). This included 11 (44%) studies reporting results for only THAs, 9 (36%) focusing on TKAs and 5 (20%) studies reporting on both THAs and TKAs.

Spacer-related complications (e.g., fracture, dislocation, exchange) were common during the interstage period with a non-weighted incidence rate of 12.7 ± 6.5% across 14 studies prior to stage 2. The rate for spacer-related complications was 10.4 ± 3.9% for THAs and 17.7 ± 8.1% for TKAs. Several studies reported on the impact of spacer design on outcomes. Jones et al. reported that molded and handmade cement spacers used in patients with THA PJIs had a greater rate of dislocation and fracture compared to antibiotic-coated prosthesis (ACP) spacers with or without a polyethylene liner [21]. Roof et al. reported that patients with TKA PJIs receiving all-cement articulating spacers underwent more reoperations after Stage 1 than patients receiving real-component spacers [46]. Lancaster et al. reported that spacer fractures occur more frequently in THA patients who have had an extended trochanteric osteotomy (ETO) [28]. 

Three studies focused on the incidence of acute kidney injury (AKI) associated with the use of antibiotic cement spacers. Dagneaux et al. reported a 45% incidence of AKI in THA patients who had pre-existing chronic kidney disease (CKD) and 14% incidence in those with no pre-existing CKD [22]. For those without pre-existing CKD, 15.4% had sustained AKI which progressed to CKD with half of these patients requiring dialysis. Geller et al. reported a 26% incidence of AKI after first-stage joint revision for the treatment of PJIs in THA and TKA patients [66]. Valenzuela et al. reported that high-dose antibiotic cement spacers used for the treatment of PJIs is an independent risk factor for AKI with 22.7% of patients developing AKI following the first stage of a planned 2-stage exchange and 3.0% of patients requiring dialysis [75]. A higher rate of AKI was observed for patients with underlying CKD. The authors recommended that efforts to minimize nephrotoxicity should be employed in revisions for PJIs when possible.

Other common complications during the interstage period and their non-weighted incidence rates reported across several studies included dislocation (6.9%) and periprosthetic fracture (4.2%). Additional procedures which were commonly required included the need for redebridement (13.9%), amputation (5.5%), and arthrodesis (5.1%) and Girdlestone (4.5%). Patients undergoing the latter three procedures resulted in them not being candidates for reimplantation as a part of the planned two-stage procedure.

While 9 (13.8%) studies reported on deaths which occurred during the interstage period, only a one of these studies specifically noted that the deaths were PJI related with a reported death rate of 7.5%.^62^ The percentage of patients dying during the interstage period for the remaining 8 studies ranged from 2 to 25% with a non-weighted average of 7.6% [18, 21, 25, 38, 47, 59, 61, 63, 70].

#### Post-reimplantation

Forty-one of the 65 (63.1%) studies provided comprehensive or selective information on complications and/or additional procedures performed following reimplantation. This included 12 (29.3%) studies reporting results for only hips, 17 (41.5%) focusing on knees and 12 (29.3%) studies reporting on both hips and knees. There were numerous reported complications and additional procedures required. Table 4 reports lists the most commonly reported complications and additional procedures required following reimplantation for these studies were identified and their non-weighted average incidence across the studies, excluding reinfections which were reported during the interstage period. Supplemental Table 3 reports detailed information on complications associated with or following the reimplantation procedure.

Thirteen (20.0%) studies reported on deaths which occurred following reimplantation. Only 4 (30.7%) of these studies specifically noted that the deaths were PJI related with an average reported death rate of 5.7% [55, 63, 64, 68]. The average percentage of patients dying following reimplantation for the remaining 9 studies was 6.0%.

### Outcomes based on MSIS reporting tool

The Musculoskeletal Infection Society (MSIS) has developed guidelines for reporting outcomes after the surgical treatment of PJIs [82]. The MSIS PJI outcomes reporting tool is organized into 4 tiers, with each tier encompassing different levels of perceived success or failure (Supplemental Table 4).

Four of the studies in the present analysis (Appendix 3) utilized the MSIS Outcomes Reporting Tool [26, 55, 59, 71]. This included one study using the tool to report outcomes for patients with THA PJIs, two studies reporting outcomes for TKA PJIs and one study reporting outcomes for both THA and TKI PJIs. Table 5 summarizes the MSIS outcomes for these 4 studies. Patients undergoing treatment for TKA PJIs had less successful outcomes compared to those being treated for THA PJIs (Table 6). This includes a lower rate of infection control when combining Tier 1 and Tier 2 (46.0% vs. 65.5%), a higher rate of reoperation and/or revision and/or spacer retention (40.6% vs. 25.2%) and a higher death rate (13.4% vs. 9.4%).

**Table 5:** Outcomes for studies using the MSIS reporting tool**

	Li K, et al. [24]	Shichman I, et al. [53]	Hartzler MA, et al. [59]	Borsinger TM, et al. [71]	Combined studies
Number of patients	205	90	134	121	550
Tier 1	122 (59.5)	19 (21.1)	49 (36.6)	59 (48.8)	249 (45.3)
Tier 2	18 (8.8)	9 (10.0)	26 (19.4)	4 (3.3)	57 (10.4)
Tier 3 Tier 3 A Tier 3B Tier 3 C Tier 3D Tier 3E Tier 3 F	50 (24.4) 3 (1.5) 2 (1.0) 4 (2.0) 3 (1.5) 6 (2.9) 32 (15.6)	50 (55.6) 2 (2.2) 14 (15.6) 0 (0.0) 15 (16.7) 13 (14.4) 6 (6.7)	41 (30.6) 3 (2.2) 7 (5.2) 6 (4.5) 11 (8.2) 7 (5.2) 7 (5.2)	38 (31.4) 0 (0.0) 5 (4.1) 6 (5.0) 14 (11.6) 6 (5.0) 7 (5.8)	179 (32.5) 8 (1.5) 28 (5.1) 16 (2.9) 43 (7.8) 32 (5.8) 52 (9.5)
Tier 4 Tier 4 A Tier 4B	15 (7.3) 5 (2.4) 10 (4.9)	12 (13.3) 2 (2.2) 10 (11.1)	18 (13.4) 9 (6.7) 9 (6.7)	20 (16.5) 13 (10.7) 7 (5.8)	65 (11.8) 29 (5.3) 36 (6.5)

*Data for each Tier reported as number of patients (%)

**Table 6:** THA vs. TKA PJI outcomes for studies using the MSIS reporting tool**

	THA PJIs [26, 71]	TKA PJIs [55, 59, 71]
Number of patients	254	296
Tier 1	147 (57.9)	68 (30.4)
Tier 2	19 (7.5)	35 (15.6)
Tier 3 Tier 3 A Tier 3B Tier 3 C Tier 3D Tier 3E Tier 3 F	64 (25.2) 3 (11.8) 2 (0.8) 6 (2.4) 9 (3.5) 7 (2.8) 37 (14.6)	91 (40.6) 5 (2.2) 21 (9.4) 6 (2.7) 26 (11.6) 20 (8.9) 13 (5.8)
Tier 4 Tier 4 A Tier 4B	24 (9.4) 12 (4.7) 12 (4.7)	30 (13.4) 11 (4.9) 19 (8.5)

*Data for each Tier reported as number of patients (%)

A separate analysis was conducted to assess the MSIS outcomes for the remaining 61 studies when the data was available (Supplemental Table 5). Many of the studies did not include data needed to categorize the outcomes into the appropriate MSIS Tiers, did not report the timing of reoperations/revisions or deaths, or only reported partial data for some of the outcomes included in the MSIS reporting tool. As a result, the data in Table 7 does not include outcomes associated with each of the sub-Tiers. There was also variability in the number of patients treated which could be assigned to each Tier.

**Table 7:** Outcomes for studies not using the MSIS reporting tool

Tier	Number of total patients treated	Number (%) in each MSIS Tier
Tier 1	475	99 (20.8)
Tier 2	1,430	508 (35.5)
Tier 3	32,373	10,236 (31.6)
Tier 4	28,780	1,363 (4.7)

There were some discrepancies between the outcomes for the studies that used the MSIS reporting tool, and the data extracted from the studies which did not use the tool. When combining Tiers 1 and 2, there were similar rates of infection control between the two groups (55.7% for studies using the MSIS tool compared to 56.3% for those not using the tool) with a combined infection control success rate of 56.2%. The overall reoperation, revision and spacer retention rate as reported for Tier 3 was also similar between the two groups (32.5% vs. 31.6%). There was a significant discrepancy between the two groups for Tier 4 with the studies using the MSIS tool reporting an overall death rate of 11.8% compared to 4.7% for those not using the tool. This is likely due to the latter often only reporting deaths during the interstage period and/or a shorter reporting time period following reimplantation.

## Discussion

While the present systematic literature review confirms that two-stage treatment of PJIs is effective for infection control and often leads to good long-term outcomes for many patients, the extended treatment duration, increased morbidity, cost, and interim complications present major challenges. While the two-stage treatment of PJIs is often considered the gold standard for effective management of chronic infections in patients with THAs and TKAs, based on the present analysis, the rate of infection control success is only 56.2%. Additionally, numerous studies have reported noteworthy challenges with this approach, as demonstrated by the high rates of reoperation, revision, spacer retention and death quantitatively reported in the 4 studies that utilized the MSIS outcomes reporting tool.

The two-stage process involves a prolonged time-period between the removal of the infected prosthesis and the reimplantation of a definitive prosthesis, often lasting several months. This interstage period is associated with a high risk of complications and morbidity as the patient waits to clear the infection prior to reimplantation. Complications related to the antibiotic spacer such as dislocation, fracture, or side effects associated with antibiotic therapy are common. Unfortunately, none of the studies in the present analysis reported the exact timing of these complications within the interstage period. This prevents the ability to identify if there was a temporal effect as to when these occurred and if a prolonged interstage period was associated with a higher rate of complications.

The two-stage treatment of PJIs also has both a positive and negative impact on patient QoL. While it offers significant long-term benefits by effectively managing the infection and improving joint function, the process is challenging and can negatively affect physical, psychological, and social well-being. The prolonged interstage period is especially associated with a decrease in both physical and mental aspects of a patient’s QoL. Carroll et al. conducted a discrete choice experiment (DCE) to quantify the surgical preferences of patients who underwent revision surgery for periprosthetic hip joint infection and compared one-stage with two-stage revision surgery [83]. In the setting of infected joint replacement, patients placed the highest value on restoration of function with this being more important than the number of operations they would have to undergo.

There were only two studies that reported patient feedback during the interstage period. Knebel et al. obtained patient feedback specific to the interstage period during two-stage procedures having patients complete a questionnaire before explantation of the infected prosthesis after confirmation of PJI, one day after explantation of the prosthesis but before antibiotic treatment, 1 to 3 days before reimplantation after 6 weeks of antibiotic treatment, and 3 months after reimplantation of a definitive prosthesis [84]. The highest mean combined anxiety and depression score was reported during the interstage treatment after prolonged antibiotic treatment. It was the only time-period when this mean score exceeded the preestablished threshold for the measurement tools utilized. The authors commented that PJI is associated with anxiety and depression that needs to be managed with psychological treatment and that the quality of life, life satisfaction, and progression anxiety of patients with PJI is comparable to those of patients with malignant diseases. Patients indicated that they were most afraid of the need for drastic medical interventions (i.e., leg amputation), the risk of reinfection, drug treatment, and being dependent on outside help. Furdock et al. separately reported on depression in patients undergoing single-stage aseptic revisions compared to those having two-stage revisions for PJIs at four different timepoints [15]. Patients undergoing two-stage revisions for PJI had significantly worse depression scores across all timepoints [85]. The percentage of patients having moderate depression was highest during the interstage period before reimplantation. The authors noted that a pre-existing diagnosis of depression was an independent predictor for clinically significant worsening of depression scores. The limited use of the MSIS outcomes reporting tool across studies and the use of differing measurement tools to obtain QoL feedback makes it difficult to compare results from many studies to each other directly.

There are several limitations for the present study. Most of the studies included in the present systematic review were retrospective and therefore do not represent a high level of evidence for the results reported. There was also a lack of consistency in the reporting of complications and additional procedures with some studies having a greater level of detail than others. There were only 5 studies which used the MSIS tool to report outcomes and none utilized CTCAE to report adverse events. Additionally, many studies which report deaths did not indicate if these were directly associated with a PJI. These limitations make it difficult to accurately compare results from one study to another and highlights the need for an improvement in the use of standardized reporting criteria and tools for future studies. Additionally, while many studies reported the duration of the interstage and overall follow-up periods, the timing of complications and procedures within these time periods was not reported, making it not possible to determine temporal effects for occurrences of these outcomes. Finally, the risk of bias and heterogeneity between studies was not assessed for the present review.

## Conclusions

While two-stage exchange arthroplasty remains the gold standard for the treatment of THA and TKA PJIs, the procedure is associated with major morbidity and often requires additional surgical procedures to address complications. The prolonged duration of the interstage period contributes to morbidity and negatively impacts patients’ QoL and increases the risk of mortality. Overall, there is a critical need for strategies to minimize the interstage period and reduce procedure-related complications. Additionally, the adoption of standardized tools and reporting criteria is essential to improve the consistency and accuracy of PJI outcome reporting, enabling better evaluation and optimization of this treatment approach.

## Data Availability

No datasets were generated or analysed during the current study.
